# Mechanical Stability and Binder Interaction in MOF Composites: An Integrated Experimental and DFT Study

**DOI:** 10.1021/acsomega.5c09981

**Published:** 2026-01-26

**Authors:** Flávia H. Silva, Mateus A. M. Paiva, Inna M. Nangoi, Maria Eduarda T. Lima, Talita V. F. da Silva, Leonã S. Flores, Raphael B. Menezes, Charlane C. Corrêa, Alexandre A. Leitão

**Affiliations:** † GPQMAP: Grupo de Pesquisa em Química dos Materiais Porosos, Departamento de Química, 28113 Universidade Federal de Juiz de Fora (UFJF), Juiz de Fora-MG 36036-900, Brazil; ‡ GFQSI: Grupo de Físico-Química de Sólidos e Interfaces, Departamento de Química, Universidade Federal de Juiz de Fora (UFJF), Juiz de Fora-MG 36036-900, Brazil; § 42506 PETROBRAS-CENPES, Cidade Universitária, Ilha do Fundão, Rio de Janeiro-RJ 21941-915, Brazil

## Abstract

An integrated study combining experimental methods and DFT simulations was undertaken to investigate the binder/MOF composites. The findings demonstrate a strong correlation between the crush strength and surface adsorption energy of methylcellulose (MC) on HKUST, ZIF-8, UiO-66­(Zr), and MIL-53­(Al)-TDC surfaces. Experimental thermogravimetric data and compressive strength measurements, combined with simulated adsorption energies, bond distances, and charge distributions, supported this relationship. DFT calculations of adsorption energy and structural analysis showed that MC/HKUST and MC/ZIF-8 composites exhibit stronger interactions with MC, consistent with their higher mechanical strength at higher temperatures. In contrast, MC/UiO-66­(Zr) and MC/MIL-53­(Al)-TDC presented weaker interactions and lower resistance under thermal stress. This study demonstrates that computational methods, when integrated with experimental techniques, can offer valuable insights into the design of mechanically stable binder/MOF composites.

## Graphical abstract

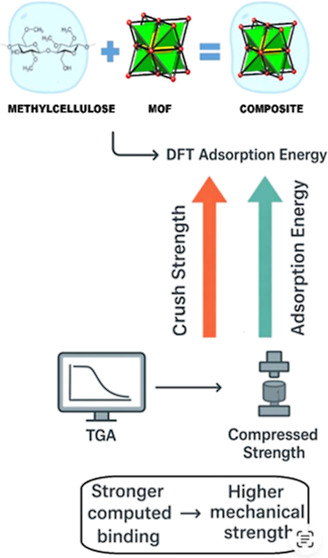

## Introduction

Metal–organic frameworks (MOFs) constitute an emerging class of porous materials characterized by exceptional porosity, high specific surface area, an abundance of active sites, and remarkable structural tunability. These properties make MOFs highly promising for various applications, including adsorption,^1,2^ separation,^3,4^ drug delivery,^5,6^ and catalysis.^7,8^ However, their practical implementation is hindered by their typical powder form, which results in poor mechanical stability, low volumetric efficiency, limited mass and heat transfer rates, challenging handling, and significant pressure drops.^9–11^ Shaping MOFs is an efficient strategy to overcome the limitations of powdered material.^9,12,13^ However, an ideal shaping method should mitigate these drawbacks without compromising the intrinsic properties of the pristine material. The most representative MOF shaping methods include granulation,^14,15^ extrusion,^16,17^ pressing,^18,19^ spray drying,^20,21^ and sol–gel method.^22,23^ Among the available shaping techniques, extrusion offers a cost-effective and technically feasible approach for large-scale production of shaped materials.^24,25^ This method prevents structural collapse, an issue commonly observed in pressing processes but typically requires the addition of a binder to achieve mechanically robust bodies.^13^ The binder promotes the aggregation of MOF powder particles and generally enhances the mechanical stability of the final product.^10,26^ The use of organic binders has shown promising results in producing mechanically robust beads while preserving MOF crystallinity and porosity.^16,27–29^ The interactions between the binder and the MOF are a key factor in promoting composites with satisfactory mechanical stability, as both the number of interactions established and their nature are related to the robustness of the final material.^30,31^ Therefore, the choice of binder for the shaping process is a fundamental aspect. Although binders have been applied to the shaping of MOFs, the formulation of these materials is typically based on an experimental trial-and-error approach.^25,32–34^ Consequently, the MOF literature still lacks studies in which this relationship is investigated in greater depth to provide a better understanding of the correlation between binder–MOF interactions and the mechanical robustness of the shaped material. Naskar et al.^35^ were pioneers in proposing the use of computational methods to evaluate the effectiveness of shaping CALF-20 with different organic binders. The authors also analyzed how the overall porosity of the CALF-20/binder composites influences the CO_2_/N_2_ selectivity, the CO_2_ sorption capacity, and the sorption kinetics. Among the binders investigated, carboxymethyl cellulose was predicted to offer a suitable balance by providing strong adhesion between the components while preserving high CO_2_/N_2_ selectivity, substantial CO_2_ uptake, and CO_2_ transport rates comparable to those of pristine CALF-20. In this work, DFT calculations were explored as a complementary tool to better understand binder–MOF interactions for four well-studied MOFs: HKUST,^36^ ZIF-8,^37^ UiO-66­(Zr),^38^ and MIL53­(Al)-TDC.^39^ Methylcellulose (MC) was used as the binder due to its economic and environmental viability^40–43^ as well as its ability to produce shaped MOF composites with high crush strength without compromising the textural properties of the MOF.^44–46^ The integration of experimental data with DFT simulations revealed a good correlation between the crush strength and the surface adsorption energy of MC on the four MOF surfaces. To reach this conclusion, the mechanical stability of extruded MC/MOF composites was evaluated through crush strength measurements and thermogravimetric analysis and then compared with the simulated results. From the simulation perspective, the interactions between the binder and the MOF surfaces were analyzed through adsorption energies, bond distances, and charge distributions. The findings presented in this work, supported by other literature references,^31,40,47^ highlight the potential of computational approaches in guiding the design and prediction of new mechanically stable MOF-based composite materials.

## Experimental Section

### Chemical Reagents

Copper­(II) nitrate trihydrate (CAS 10031-43-3), zinc nitrate hexahydrate (CAS 10196-18-6), trimesic acid (CAS 554-95-0), terephthalic acid (CAS 100-21-0), 2-methylimidazole (CAS 693-98-1), 2,5-thiophenedicarboxylic acid (CAS 4282-31-9), and methylcellulose 400 cP (CAS 9004-67-5) were purchased from Sigma-Aldrich. Zirconyl chloride octahydrate (CAS 13520-92-8) and aluminum chloride hexahydrate (CAS 7784-13-6) were purchased from Neon Reagents. *N*,*N*-Dimethylformamide (DMF) (CAS 68-12-2) and ethanol (EtOH) (CAS 64-17-5) were purchased from Neon Reagents. Anhydrous methanol (MeOH) (CAS 67-56-1) was purchased from FMaia. All of the materials were analytical grade and used without further purification.

### Preparation of MOFs

#### HKUST

HKUST was synthesized according to Khoshhal et al.;^48^ first copper­(II) nitrate trihydrate (6.0115 g, 0.0249 mol) and trimesic acid (4.0040 g, 0.0191 mol) were added to a 250 mL round-bottom flask containing DMF as the solvent (250 mL). The mixture was sonicated for 30 min. The solution was heated at 87 °C in an oil bath at reflux conditions at atmospheric pressure for 16 h. After this stage, the product was washed once with DMF (150 mL) at 80 °C and twice with EtOH (100 mL) at 70 °C. Therefore, 240 mL of a mixture of deionized water and EtOH (volume ratio of 1:5) and the solid were placed on a magnetic stirrer and stirred for 24 h. Then, the solid was isolated by filtration, washed twice with EtOH (100 mL) at 70 °C, and dried at 110 °C for 24 h.

#### ZIF-8

The synthesis of ZIF-8 was carried out according to the methodology described in Coronas et al.^49^ with adaptations. 2-Methylimidazole (6.4890 g, 0.0790 mol) was dissolved in 200 mL of anhydrous MeOH. Zinc nitrate hexahydrate (2.9330 g, 0.0099 mol) was dissolved in 200 mL MeOH and was rapidly poured into the ligand solution. After 1 h of stirring at room temperature, a white powder was obtained, which was separated by centrifugation at 4790 G for 5 min. The powder was purified by washing twice with MeOH and dried at 70 °C for 12 h.

#### UiO-66­(Zr)

UiO-66­(Zr) was synthesized based on the method reported by Ragon et al.^50^ Zirconyl chloride octahydrate (6.4450 g, 0.0200 mol) was added to a 250 mL round-bottom flask followed by 40 mL of DMF (solvent) and 9.78 mL of HCl 32% v/v (modulator). This mixture was sonicated for 20 min to ensure the complete dissolution of the metallic precursor. Finally, terephthalic acid (3.3226 g, 0.0200 mol) was added, and the bottom flask was put in an oil bath at 120 °C under reflux conditions at atmospheric pressure for 24 h. The obtained solid was filtered, washed twice with EtOH (20 mL), and air-dried. It was then transferred to a beaker with 50 mL of EtOH and left undisturbed for 24 h. The solvent was carefully removed and replaced with 50 mL of fresh EtOH twice more. The washed solid was isolated by filtration and dried at 125 °C for 24 h.

#### MIL-53­(Al)-TDC

MIL-53­(Al)-TDC was synthesized following the method described by Tannert et al.^51^ Aluminum chloride hexahydrate (4.8286 g, 0.0200 mol), 2,5-thiophenedicarboxylic acid (3.4432 g, 0.0200 mol), DMF (16 mL), and H_2_O (64 mL) were added to a round-bottom flask and coupled to reflux in an oil bath at 135 °C for 24 h. The obtained white solid was filtered, washed twice with H_2_O (60 mL), and then stirred in H_2_O (170 mL) at room temperature for 24 h. Subsequently, the solid was filtered and washed twice with EtOH (50 mL). The solid was dried at 150 °C for 24 h.

### Composite Preparation and Extrusion Procedure

The MOF composites were prepared from powdered MOFs synthesized as detailed above, using 5 wt % of MC relative to the mass of the activated MOF at 120 °C for 24 h. Initially, the dry mixture of MOF and binder was macerated for 10 min. Then, the maceration process was accompanied by adding an EtOH/H_2_O solution 50/50% (v/v) dropwise at a ratio described in Table S1, for 5 min, to obtain a homogeneous viscous paste. The paste was transferred into a 5 mL plastic syringe, and the plunger was pressed to extrude it into a cylindrical form, which was cut to produce 2–4 mm long granules. Finally, the granules were dried in an oven at 100 °C for 24 h. The specific masses used for the preparation of each granule are presented in Table S1.

### Characterization

Powder X-ray diffraction (PXRD) patterns of the materials were recorded using a Bruker D8 ADVANCE DaVinci with Bragg–Brentano θ-θ geometry and Cu–Kα radiation (λ = 1.54056 Å). Crystal structures were visualized, and diffraction patterns were simulated using Mercury (CCDC, Cambridge, UK) based on CIF files. The LynxEye detector operated at 40 kV and 40 mA, with data collected over a 2θ range of 5° to 50°. Thermal analyses (TGA; DTG curves derived thereof) were performed on a TGA 5500 analyzer (TA Instrument), and samples were heated from 30 to 800 °C at a rate of 10 °C·min^–1^ under a nitrogen atmosphere. Nitrogen adsorption/desorption isotherms were obtained at 77 K using a Micrometrics TriStar 3000 surface area and porosity analyzers. Specific surface areas were calculated using the Brunauer–Emmett–Teller (BET) method. Prior to analysis, samples were activated by heating at 120 °C under vacuum.

### Crush Strength Measurements

The mechanical stability of the extruded samples was analyzed through crush strength measurements. This technique enables determination of the force that an individual granule can withstand until the first fracture during a compression process. The measurements were carried out on a Nova Ética 298 DGP digital tablet tester with longitudinal compression, as shown in Figure S1. The crush strength value for each sample was determined as the average of the crushing strength of 20 individual extrudates.

### Computational Methodology

The structural models for all MOFs, MIL-53­(Al)-TDC,^39^ UiO-66­(Zr),^50^ ZIF-8,^52^ and HKUST,^53^ were taken from the literature and further optimized using the methodology described below. All structures, including bulk and surfaces models, were performed using the Quantum ESPRESSO package which implements DFT under periodic boundary conditions.^54–56^ Generalized gradient approximation (GGA-PBE)^57^ was employed for the exchange correlation functional, along with Grimme D3 dispersion correction.^58^ The ion cores of all atoms were treated using the projector augmented wave (PAW) method.^59^ The Kohn–Sham states were expanded in a plane-wave basis set with kinetic cutoff energy of 60, 60, 70, and 40 Ry for MIL-53­(Al)-TDC, UiO-66­(Zr), ZIF-8, and HKUST, respectively. The Methfessel-Paxton smearing technique was used with a broadening of 0.005 Ry to smooth the Fermi distribution.^60^ A Monkhorst–Pack mesh of 4 × 4 × 4 was used for MIL-53­(Al)-TDC, while calculations for all other structures were performed at the Γ-point.^61^ All parameters were selected based on convergence tests for each structure, and the relative ion positions were relaxed until all force components were smaller than 0.001 Ry Bohr^–1^.

After bulk optimization, each MOF surface was generated using the BURAI 1.3.2 software.^62^ Surface models were generated for each unique Miller index (*hkl*) with a vacuum spacing of 15 Å. Cleavage planes were selected according to the most probable fracture sites, typically located near the metal centers when applicable. The resulting broken bonds were passivated by adding water molecules and reoptimized. The two surface models with the lowest energies, as determined from eq 1, were selected for subsequent adsorption simulations (Table 3).

1 $$\varepsilon =\frac{E_{surface}-NE_{bulk}-NE_{H_{2}O}}{2A}$$

where *E*_surface_ is the DFT energy of the surface, *E*_bulk_ represents the bulk energy before surface creation, and $E_{H_{2}O}$ is the energy of a water molecule. *N* is the representative number of water molecules or bulk units used for surface creation. The smaller the value of ε, the greater the energetic stability of the surface, as it indicates a lower energy state relative to its isolated components.

Afterward, a methylcellulose fragment (Figure S4) was inserted at the center of the surface, and the structure was reoptimized. The charge distribution between the MOF surface and the methylcellulose molecule was analyzed using charge density difference plots, as defined by eq 2.

2 $$\Delta \rho \left(r\right)=\rho {\left(r\right)}_{tot}-\left(\rho {\left(r\right)}_{surface}+\rho {\left(r\right)}_{MC}\right)$$

where Δρ­(*r*) represents the charge density difference, ρ­(*r*)_tot_ is the total charge density of the combined system, ρ­(*r*)_surface_ corresponds to the charge density of the bare surface, and ρ­(*r*)_MC_ is the charge density of the isolated methylcellulose fragment. The adsorption energy was calculated using eq 3.

3 $$\Delta E_{ads}=E_{tot}-\left(E_{surface}+E_{MC}\right)$$

where *E*_tot_ is the energy of the whole system (MC/MOF), *E*_surface_ is the energy of the bare surface, and *E*_MC_ is the energy of the isolated methylcellulose.

## Results and Discussion

### Experimental Characterization

Since the shaping methodology must preserve the intrinsic properties of MOFs, including their crystalline structure and textural features, the first characterization technique employed was powder X-ray diffraction (PXRD). This analysis was conducted on both the MOF powders and their corresponding shaped composites to assess whether the extrusion process affected the crystal structure. The resulting diffractograms (Figure 1) did not show significant differences, indicating that the extrusion process did not affect the crystalline integrity of the MOFs.

**Figure 1:**
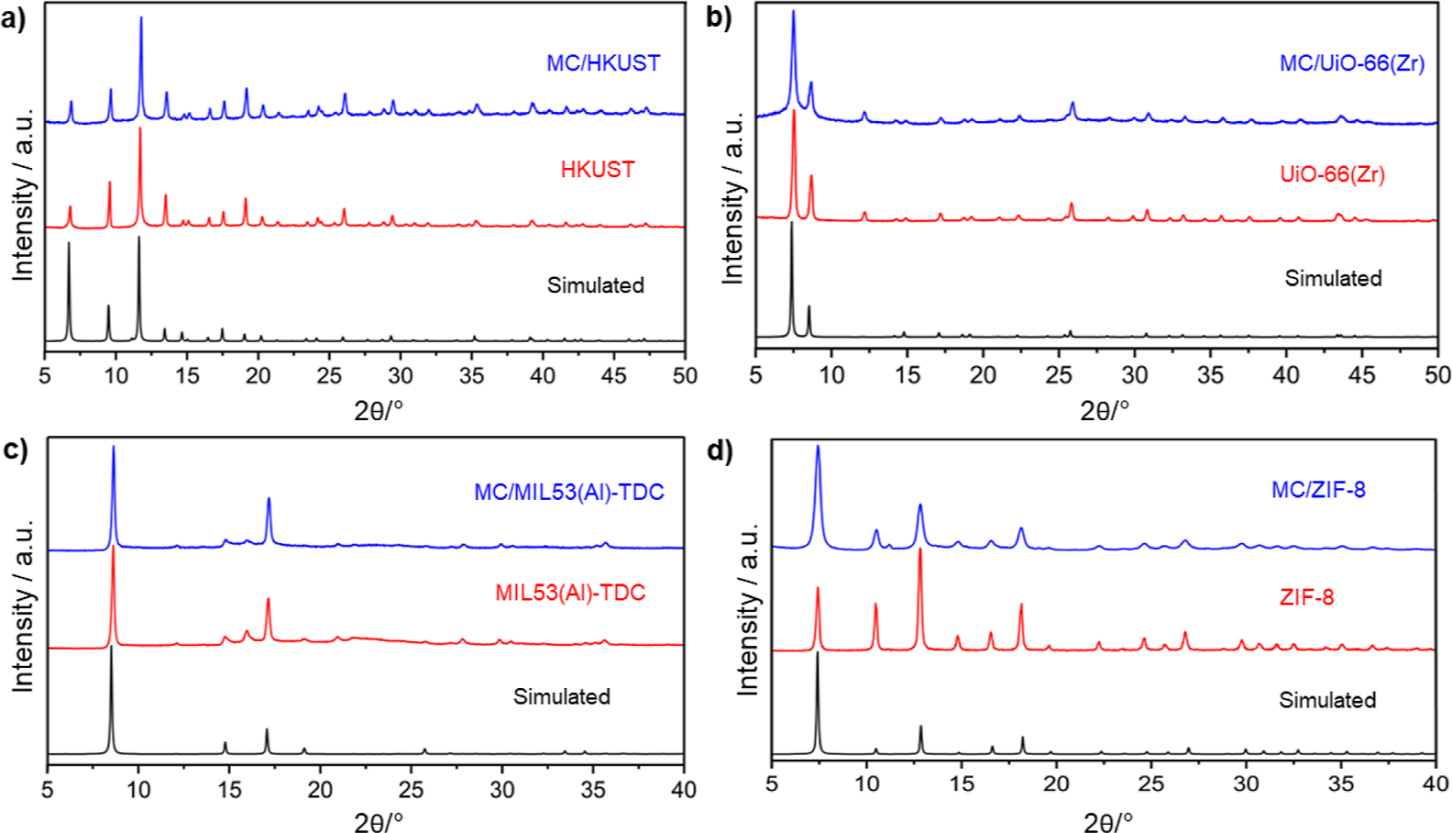
Simulated PXRD patterns derived from crystallographic data (black) and experimental PXRD patterns of pristine MOF powders (red) and their corresponding extruded composites (blue): (a) HKUST; (b) UiO-66­(Zr); (c) MIL-53­(Al)-TDC; and (d) ZIF-8.

The impact of extrusion on the specific surface area of the MOFs was evaluated through nitrogen adsorption/desorption measurements. The isotherms for both powdered samples and extrudates are shown in Figure S2. Specific surface area values, calculated by using the Brunauer–Emmett–Teller (BET) equation, along with the corresponding area reductions caused by the shaping process, are presented in Table 1. For all extrudates, the reduction in specific surface area was negligible, demonstrating that the extrusion process did not significantly compromise the porosity of the MOFs.

**Table 1:** Specific Surface Areas and Corresponding Area Reduction of Pristine and MC/MOF Composites

material	specific surface area (m^2^·g^–1^)	area reduction (%)
UiO-66(Zr)	1142	8.49
MC/UiO-66(Zr)	1045	
MIL-53(Al)-TDC	815	7.12
MC/MIL-53(Al)-TDC	757	
HKUST	1079	8.99
MC/HKUST	982	
ZIF-8	1097	5.01
MC/ZIF-8	1042	

Crush strength measurements were conducted to assess the mechanical stability of the extruded composites. The crush strength data, presented in Table 2, indicate that the extruded MOFs exhibit crush strength values comparable to those of various commercial adsorbents (Table S2), underscoring their potential for applications requiring mechanical robustness. The binder content used in the formulation (5 wt %) was sufficient to provide adequate crush strength to the powdered MOFs without compromising their textural properties.

**Table 2:** Crush Strength for the Extrudates Produced in This Work (Activated at 100 °C)

material	form	size (mm)	crush strength (*N*)[Table-fn t2fn1]
MC/UiO-66(Zr)	cylinder	2.0–3.0	14.6
MC/MIL-53(Al)-TDC	cylinder	2.0–3.0	20.4
MC/ZIF-8	cylinder	2.0–3.0	32.6
MC/HKUST	cylinder	2.0–3.0	33.7

b Average value calculated from 20 single particle crush strength measurements.

Thermogravimetric analysis (TGA) was carried out to evaluate the thermal stability of the extrudates. The thermogravimetric curves and their derivatives (DTG) are shown in Figure 2. For all samples, an initial weight loss was observed due to the presence of adsorbed water molecules, followed by MOF decomposition at temperatures above 350 °C. For the MC composites, an additional weight loss was detected in the TGA curves with shoulders around 250–300 °C. These features are clearly visible in the DTG curves and are not observed in the powdered MOF samples, indicating an intermediate decomposition step likely associated with the MC pyrolysis. Finally, the decomposition temperature of the MOFs was not compromised by the composite formulation.

**Figure 2:**
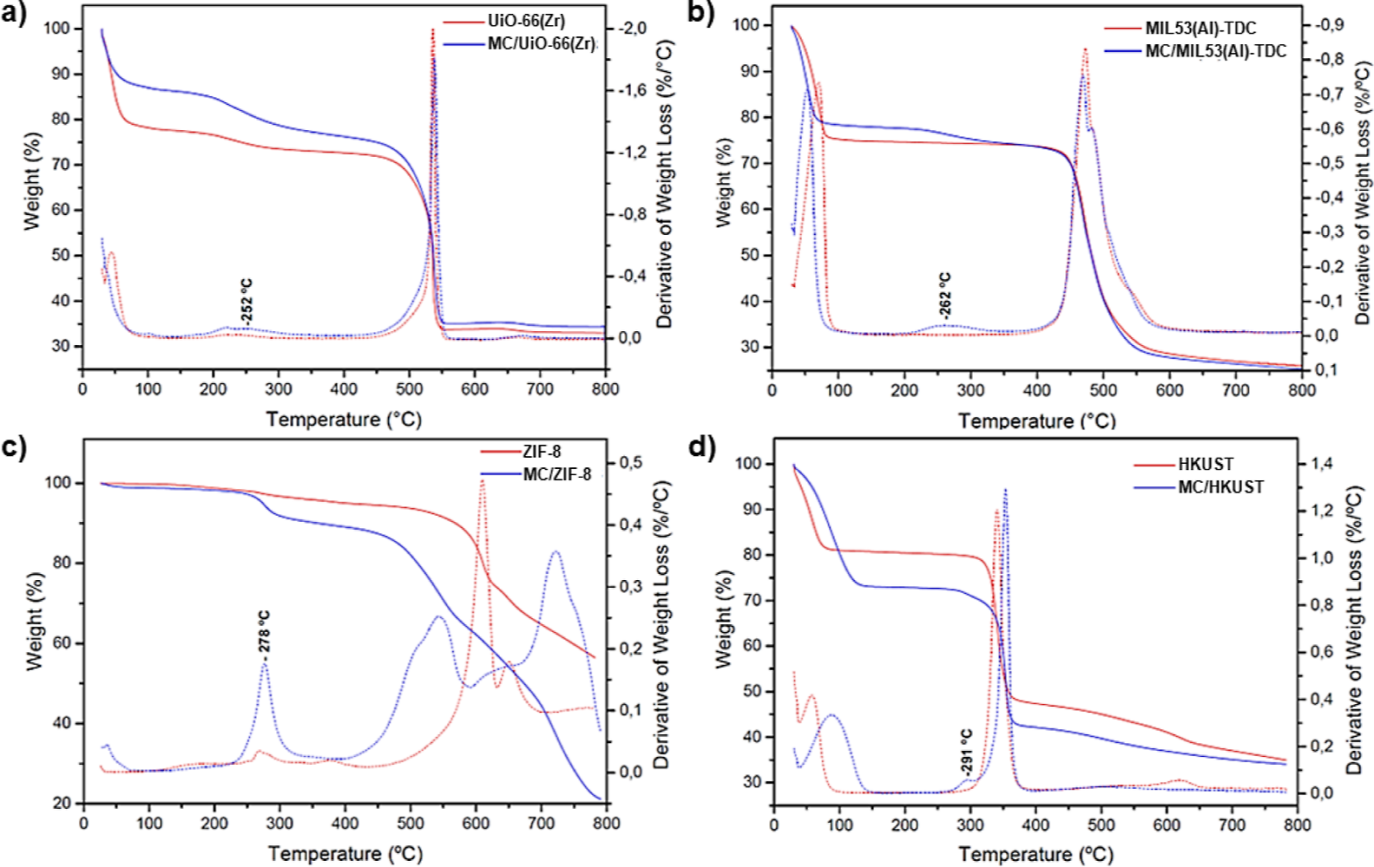
TGA (solid lines) and DTG (dotted lines) curves obtained before and after composite formulation: (a) UiO-66­(Zr) (red) and MC/UiO-66­(Zr) (blue); (b) MIL-53­(Al)-TDC (red) and MC/MIL-53­(Al)-TDC (blue); (c) ZIF-8 (red) and MC/ZIF-8 (blue); and (d) HKUST (red) and MC/HKUST.

The MC decomposition temperatures observed in MOF composites were lower than those observed for pure MC (Figure S3), consistent with previous reports on other MOF–polymer systems.^46,63^ This effect can be attributed to the catalytic role of the MOF metal centers in promoting polymer degradation.^63–65^ Furthermore, the DFT calculations presented below reveal a correlation between MC adsorption energies and the decomposition temperatures of the composites, indicating that stronger interactions between the polymeric binder and the MOF surface enhance the thermal stability of the material. The data obtained from DFT calculations are in good agreement with the TGA analyses.^66^ A similar trend has also been observed in studies of polymer matrices coated with MOFs.^67–69^

### Simulation: Structural and Energetic Analyses

The electronic energy of MOFs surfaces was calculated and chosen according to eq 1, and slab models, with their respective ε values, are reported in Table 3.

**Table 3:** Theoretical Surface Energies (in kJ·mol^–1^ Å^–2^) for MIL-53­(Al)-TDC, UiO-66­(Zr), ZIF-8, and HKUST

MIL-53(Al)-TDC	ε	UiO-66(Zr)	ε	ZIF-8	ε	HKUST	ε
(100)	–0.52	(111)	1.28	(111)	1.14	(110)	1.13
(001)	0.70	(011)	1.65	(110)	1.42	(001)	1.50
(011)	1.23	(001)	2.20	(100)	2.48	(111)	1.56
(010)	1.38			(001)	2.66		
(101)	1.87			(010)	2.65		
(111)	2.25			(011)	2.87		
(110)	2.84			(101)	4.08		

After identifying the two surfaces with the lowest ε values, a fragment of MC polymer (Figure S4) was placed on top of each surface and the combined systems were fully optimized. The interactions between MC and MOF surfaces were analyzed in terms of adsorption energies (eq 3), bond distances, and charge density difference (eq 2). The calculated adsorption energies are listed in Table 4 and, in general, the values follow the same trend as the crush strength experiment (Table 2). The obtained negative values of adsorption energies demonstrate that the MC has a strong interaction with the studied MOFs surfaces.

**Table 4:** Adsorption Energies, in kJ·mol^–1^, of Methylcellulose Fragment on Selected Crystallographic Planes of MOFs

MOF	(*hkl*) planes	adsorption energy
MC/MIL-53(Al)-TDC	(001)	–105.42
MC/MIL-53(Al)-TDC	(100)	–102.83
MC/UiO-66(Zr)	(011)	–150.83
MC/UiO-66(Zr)	(111)	–74.31
MC/ZIF-8	(110)	–183.21
MC/ZIF-8	(111)	–145.06
MC/HKUST	(001)	–191.20
MC/HKUST	(110)	–100.21

The differences in the adsorption energies can be explained by the specific interactions of MC with the hydroxyl groups on the MOF surfaces. The structures with the highest adsorption energies, MC/HKUST and MC/ZIF-8, i.e., those with stronger interactions, are the ones that form strong to moderate hydrogen bonds^70^ between MC and MOF surface (Figures S9–S12). On the other hand, when there is only a single anchoring point, as observed for UiO-66­(Zr) (Figure S7) and HKUST (Figure S12), the adsorption energy decreases. Charge density difference (CDD) plots (Figures S5 and S12) illustrate the electronic interactions between the polymer fragment and the MOF surfaces. In HKUST, for instance, a clear accumulation charge is observed in the oxygen atom directly coordinated to the metal center coming from the hydroxyl group of MC (Figure 3a and b). On the (001) HKUST surface, MC establishes two hydrogen bonds, whereas on the (110) surface, interaction occurs at only one anchor point. This difference in the number and strength of hydrogen bonds accounts for the variation in the adsorption energy among these surfaces. A similar charge transfer pattern is found on the (110) surface of ZIF-8 (Figure 3c), which exhibits the second highest adsorption energy. In the case of MC interaction with the most stable MIL-53­(Al)-TDC surfaces, the interactions can be considered as mainly electrostatic, reflecting a weaker crush strength shown in the experimental results.

**Figure 3:**
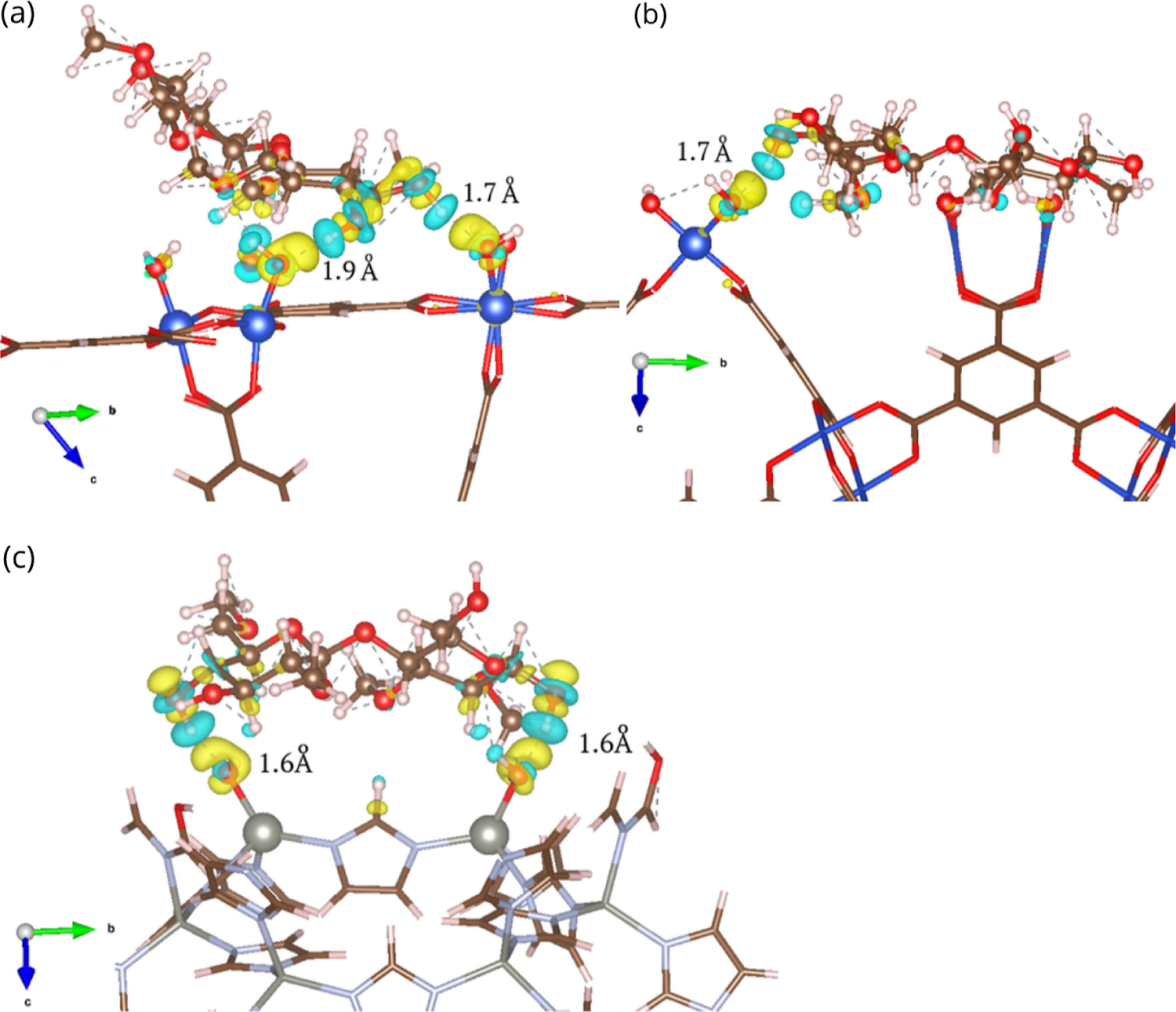
Charge density difference between MC and (001) surface (a) and (110) surface (b) of HKUST and (110) surface of ZIF-8 (c). The isosurface contour is 0.003 e/Bohr^3^. Yellow and cyan regions represent charge accumulation and depletion between the molecules, respectively. Cu, blue; Zn, dark silver; N, light silver; C, brown; O, red; H, white.

Overall, the results demonstrate that the MOF surface significantly influences the strength of interaction with methylcellulose. A slight deviation from the overall trend was observed for UiO-66­(Zr) and MIL53­(Al)-TDC. The synthesis methodology may influence crystal growth along less stable, yet kinetically favored directions, resulting in surface terminations with reduced capacity to interact with the binder.^71–73^ Likewise, our simulations demonstrated that the (001) surface of UiO-66­(Zr) exhibits a much weaker interaction with MC. Crystal growth in this direction results in a surface that is less favorable for MC adsorption, which may, in turn, reduce the crush strength of the resulting composite^74^ Although surface formation may render the MC/MOF interaction less favorable from an energetic standpoint, the resulting composites still demonstrate good mechanical resistance, as confirmed experimentally. Furthermore, it is worth noting that the composite preparation can also impact the crush strength results.^65,75^

### Mechanical and Structural Stability under Thermal Conditions

To evaluate the stability of the MC/MOF extruded composites produced in this study, we investigated their mechanical and structural behavior after the thermal treatment. The extrudates were exposed to temperatures higher than those applied during the extrusion process (100 °C) in air for 24 h. Although the crystalline structure was preserved in all samples treated at 150 °C (Figure 4), a reduction in crush strength was observed (Table 5), with smaller losses for the MC/HKUST and MC/ZIF-8 composites.

**Figure 4:**
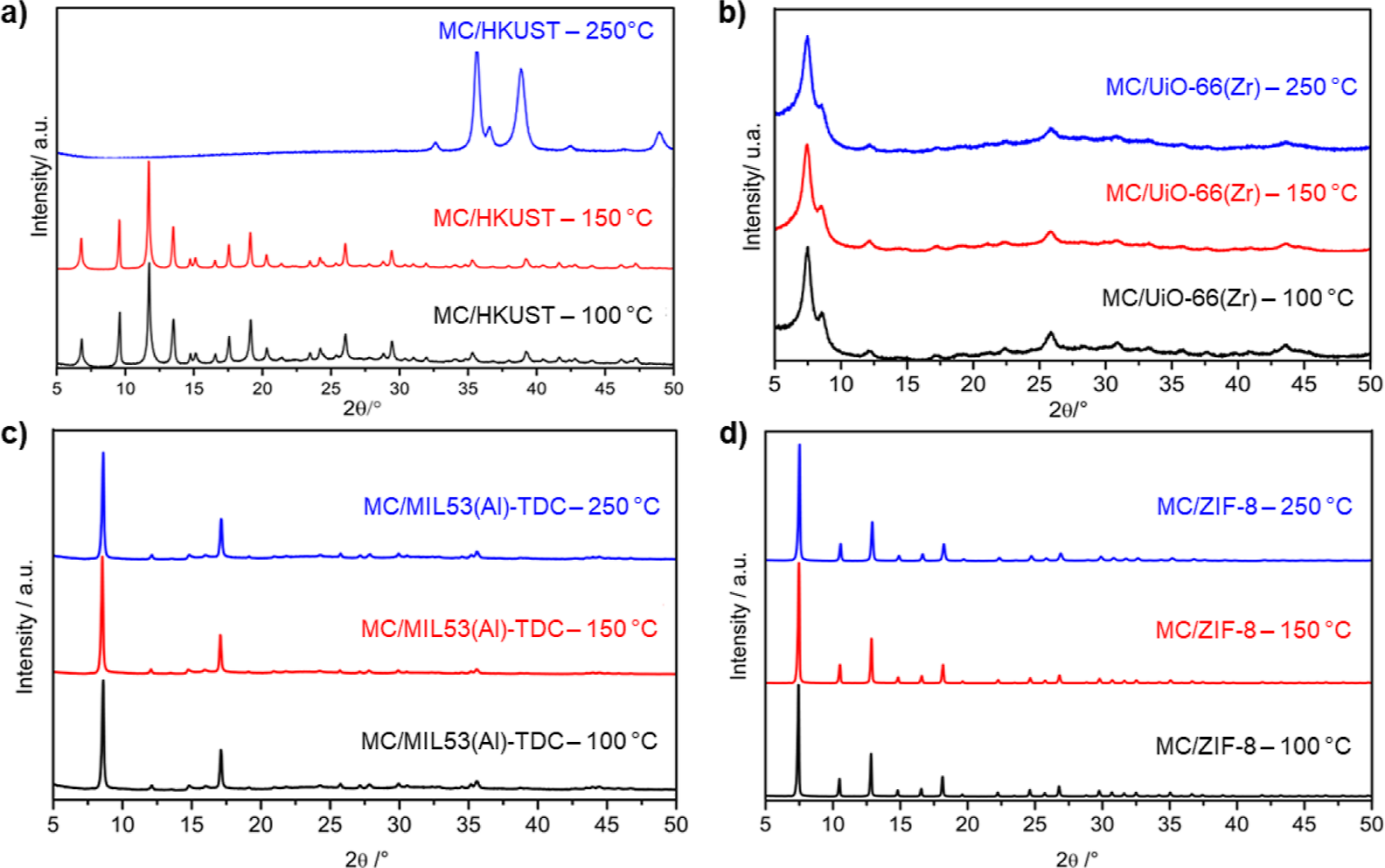
Experimental PXRD patterns of extruded composites after heat treatment in air at 100 °C (black), 150 °C (red), and 250 °C (blue): (a)­MC/HKUST; (b) MC/UiO-66­(Zr); (c) MC/MIL-53­(Al)-TDC; and (d) MC/ZIF-8.

**Table 5:** Crush Strength of the Extrudates after Exposure to Various Thermal Treatments in Air for 24 h

sample	crush strength at 100 °C (*N*)	crush strength at 150 °C (*N*)	crush strength at 250 °C (*N*)
MC/UiO-66(Zr)	14.6	4.6	3.8
MC/MIL-53(Al)-TDC	20.4	6.4	4.1
MC/ZIF-8	32.6	30.8	2.4
MC/HKUST	33.7	30.2	–

DFT calculations indicated that MC interacts with the MOFs primarily through hydrogen bonding. When thermal treatment is applied, these interactions can be disrupted, leading to a reduction in the crush strength. Furthermore, the mechanical stability of the composites can be affected by elevated temperatures due to the reorganization of MC chains and/or the weakening of hydrophobic interactions, both of which may contribute to a reduction in crush strength.^76^ The greater number of interactions and higher MC adsorption energy values predicted by DFT for the MC/HKUST and MC/ZIF-8 composites may account for the minor changes observed in their mechanical stability after heating to 150 °C.

Upon heating to 250 °C, the MC/HKUST composite undergoes complete decomposition, yielding a black powder (Figure 5). PXRD analysis confirms that the resulting material corresponds to CuO (Figure 4a).^77,78^ Although MC/UiO-66­(Zr), MC/MIL-53­(Al)-TDC, and MC/ZIF-8 retain their crystalline structure and granular morphology up to 250 °C, a pronounced reduction in mechanical strength is observed. TGA results after heat treatment indicate that prolonged exposure to elevated temperatures in air leads to thermal degradation of MC. For all extruded composites, the thermogravimetric curves show the absence of MC-related weight loss after heating at 250 °C, whereas this feature is clearly present in samples treated at 100 and 150 °C, as shown in Figure 6.

**Figure 5:**
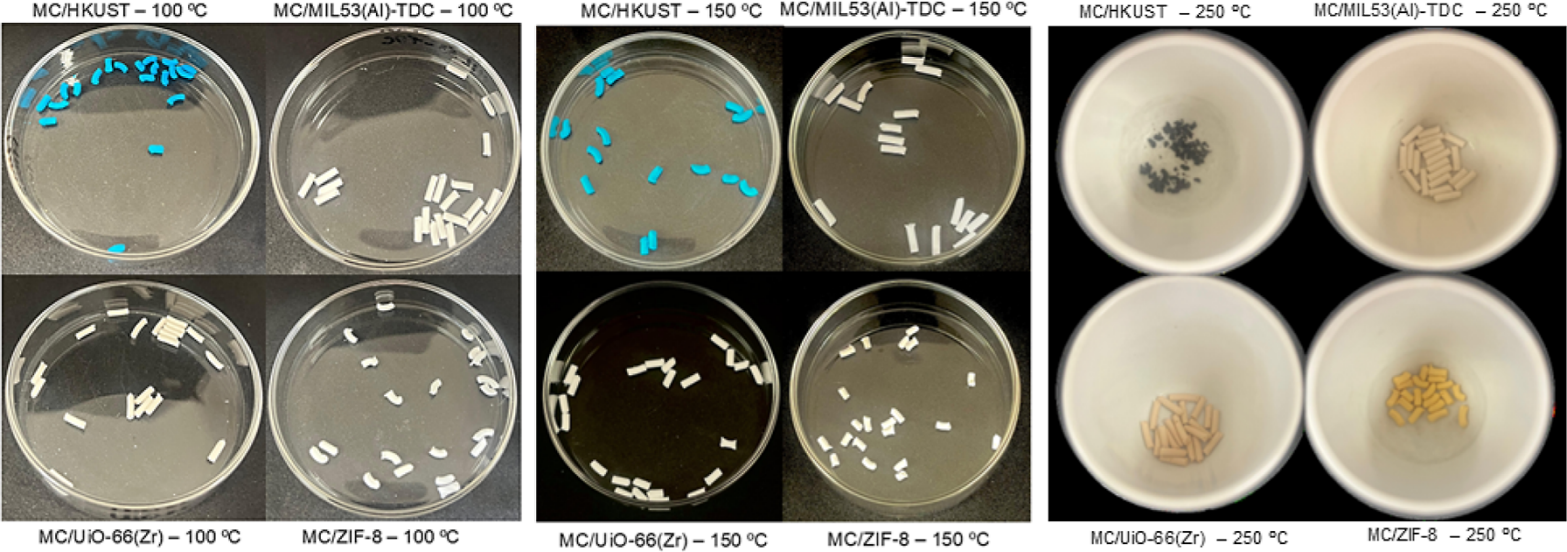
Photograph of extruded composites after heat treatment at 100 °C, 150 °C, and 250 °C. Legend in the picture. All photographs were taken by one of the authors.

**Figure 6:**
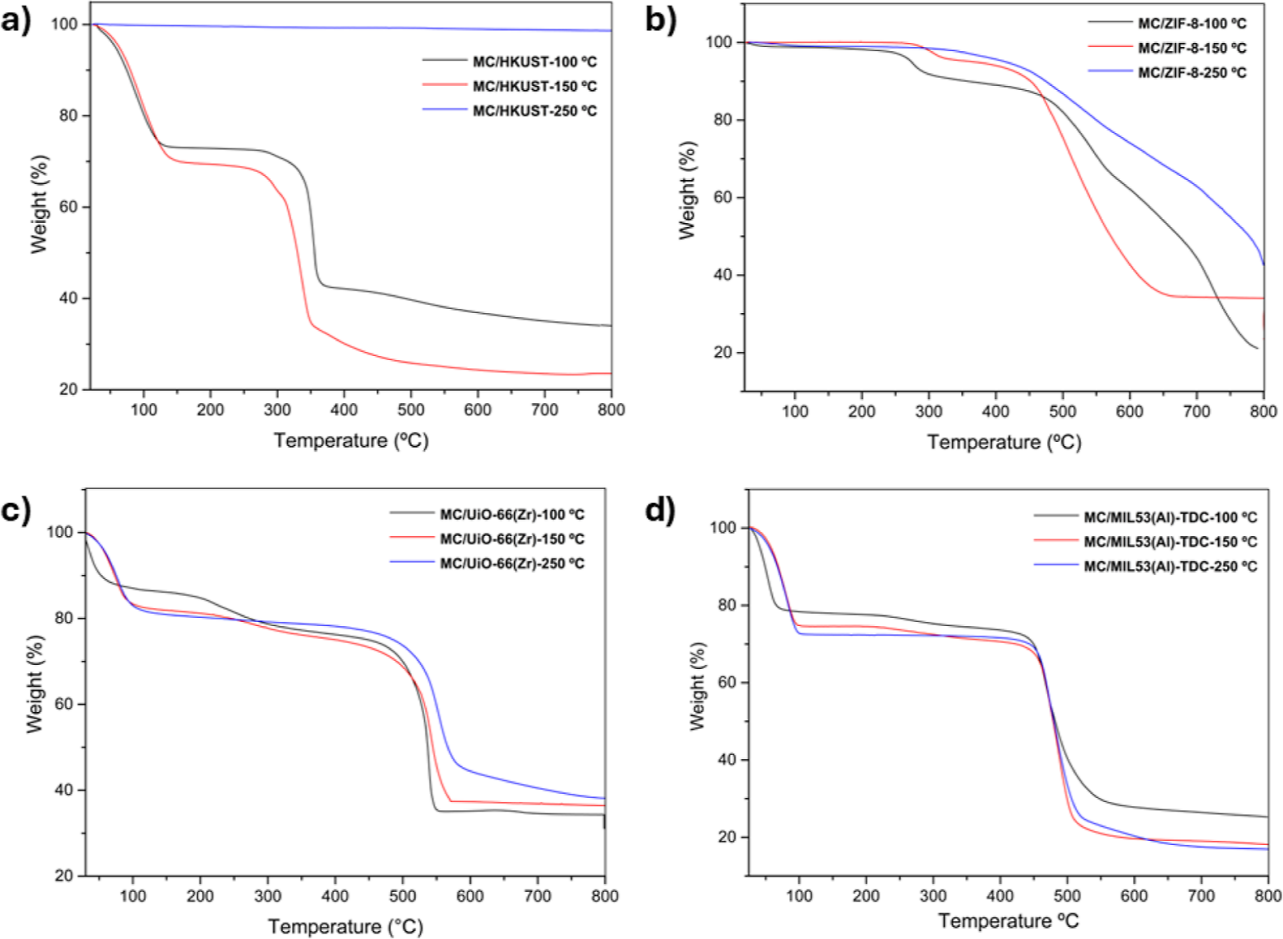
TGA curves obtained after heat treatment in air at 100 °C (black), 150 °C (red), and 250 °C (blue): (a) MC/HKUST; (b) MC/ZIF-8; (c) MC/UiO-66­(Zr); and (d) MC/MIL-53­(Al)-TDC.

So far, we can conclude that all MC/MOF extruded composites evaluated here are suitable for applications operating at temperatures up to 100 °C, while MC/HKUST and MC/ZIF-8 remain stable at temperatures up to 150 °C, suggesting their potential suitability for applications within this temperature range.

This work evaluated the interactions and mechanical stability under dry conditions. For practical applications, it is important to consider the effect of ambient humidity, as water molecules can compete for the adsorption sites and hydrogen bonds identified as crucial for MC adhesion. The extensive literature on the hydrolytic stability of these MOFs^9,79,80^ suggests that while HKUST would undergo structural degradation, ZIF-8, UiO-66­(Zr), and MIL-53­(Al)-TDC would likely maintain their integrity, although the strength of the MC/MOF interface could be modulated.^9,79,80^ Consistent with this expectation, PXRD patterns (Figure S13) collected for the composites more than six months after their preparation and activation at 100 °C demonstrated the preservation of the crystalline structures. The improved air stability of MC/HKUST for a period of one month has been previously reported;^45^ here, stability over a longer period is confirmed. Despite these favorable results, the strength of the MC/MOF interface may be modulated by the presence of water. Therefore, future investigations addressing the crush strength of the composites after controlled humidity exposure are recommended to complement the present findings.

### Conclusion

Four extruded MC/MOF composites were synthesized, exhibiting average crush strength values on the same order of magnitude as those of commercial adsorbents. Overall, the experimental crush strength values were consistent with the MC/MOF adsorption energy values obtained from the DFT calculations. The simulations revealed that MC/HKUST and MC/ZIF-8 composites establish stronger interactions with MC through a hydrogen-bond network, and these composites correspondingly exhibited higher crush strength values. Moreover, the MC/UiO-66­(Zr) and MC/MIL-53­(Al)-TDC composites displayed lower mechanical resistance, in agreement with the DFT results. The computational findings provided deeper insight into the interaction between MC and the studied MOFs, suggesting that DFT calculations could be a valuable tool for guiding the design of mechanically stable binder/MOF composites.

The mechanical and structural stability of the extruded composites was evaluated after thermal treatment, which is a condition commonly encountered in real-world applications. The results indicated that all composites are suitable for processes operating at temperatures up to 100 °C. Only MC/HKUST and MC/ZIF-8 remained stable at temperatures up to 150 °C. DFT results indicate that methylcellulose (MC) interacts with MOFs predominantly through hydrogen bonding, which can be weakened upon heating, leading to a decrease in the mechanical strength. These findings suggest that the MC/MOF composites produced in this work have the potential for a variety of applications within these temperature ranges.

## Supplementary Material



## Notes

Photographs illustrating a crush strength measurement; nitrogen isotherms of powdered MOFs and their extruded composites; TGA curve and DTG curve of methylcellulose; optimized methylcellulose structure; table of MOF composite preparation conditions; crush strength of commercial materials; charge density difference plots; and experimental PXRD patterns of extruded composites activated in air at 100 °C and stored for more than six months (PDF)
