# Supplementary material for: Mechanical Stability and Binder Interaction in MOF Composites: An Integrated Experimental and DFT Study
Source: ACS Omega. 2026 Jan 26;11(5):7898–907. doi: 10.1021/acsomega.5c09981 (PMC12902986; doi:10.1021/acsomega.5c09981)
Supplement: Supplementary file 1 [file ao5c09981_si_001.pdf]

Supporting Information

Mechanical Stability and Binder Interaction in MOF Composites: An Integrated Experimental and DFT Study

Flávia H. Silva,† Mateus A. M. Paiva,† Inna M. Nangoi,‡ Maria Eduarda Toledo Lima, Talita V. F. da Silva, Leonã S. Flores, Raphael B. Menezes, Charlane Q. Corrêa, and Alexandre A. Leitão‡

†GPQMAP: Grupo de Pesquisa em Química dos Materiais Porosos, Departamento de Química, Universidade Federal de Juiz de Fora (UFJF), Juiz de Fora-MG, 36036-330, Brazil

‡GFQSI: Grupo de Físico-Química de Sólidos e Interfaces, Departamento de Química, Universidade Federal de Juiz de Fora (UFJF), Juiz de Fora-MG, 36036-330, Brazil

PETROBRAS-CENPES, Cidade Universitária, Ilha do Fundão, Rio de Janeiro-RJ, 21941-915, Brazil

E-mail: inna.nangoi@estudante.ufjf.br

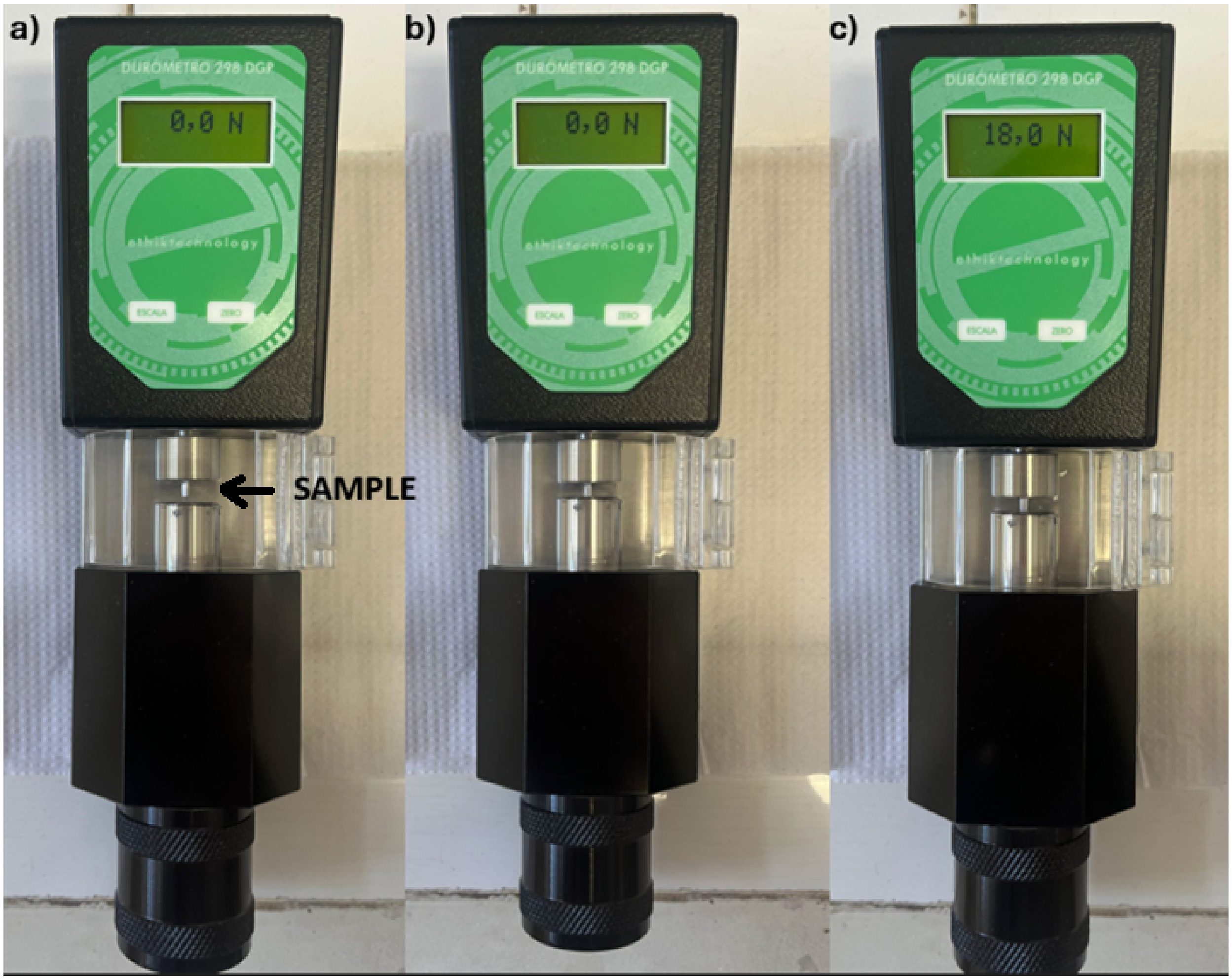

Figure S1: Photographs illustrating a crush strength measurement before the compaction process (a), at the beginning of the test (b), and after granule fracture (c).

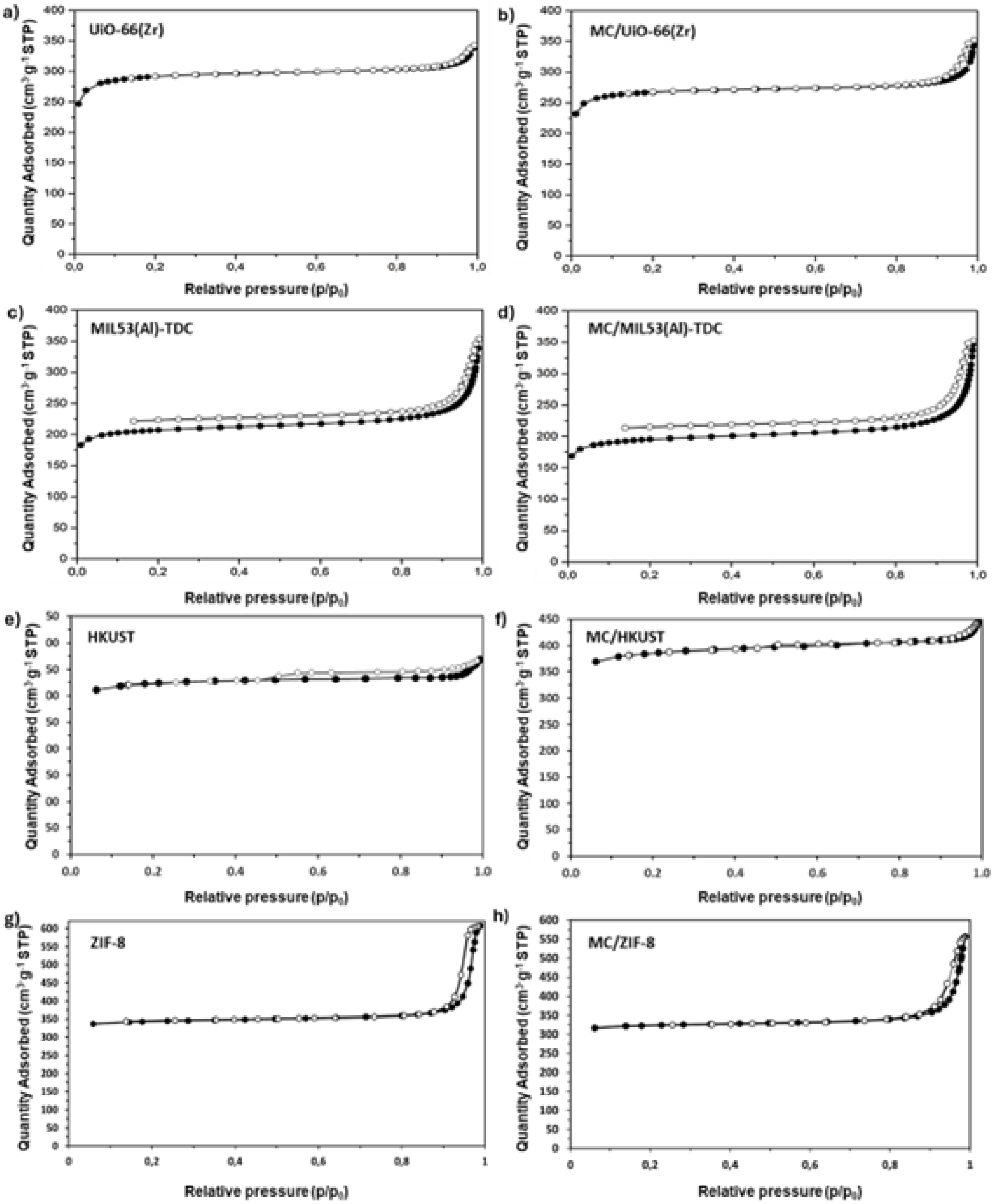

Figure S2: Nitrogen adsorption/desorption isotherms of powdered MOFs and their extruded composites.

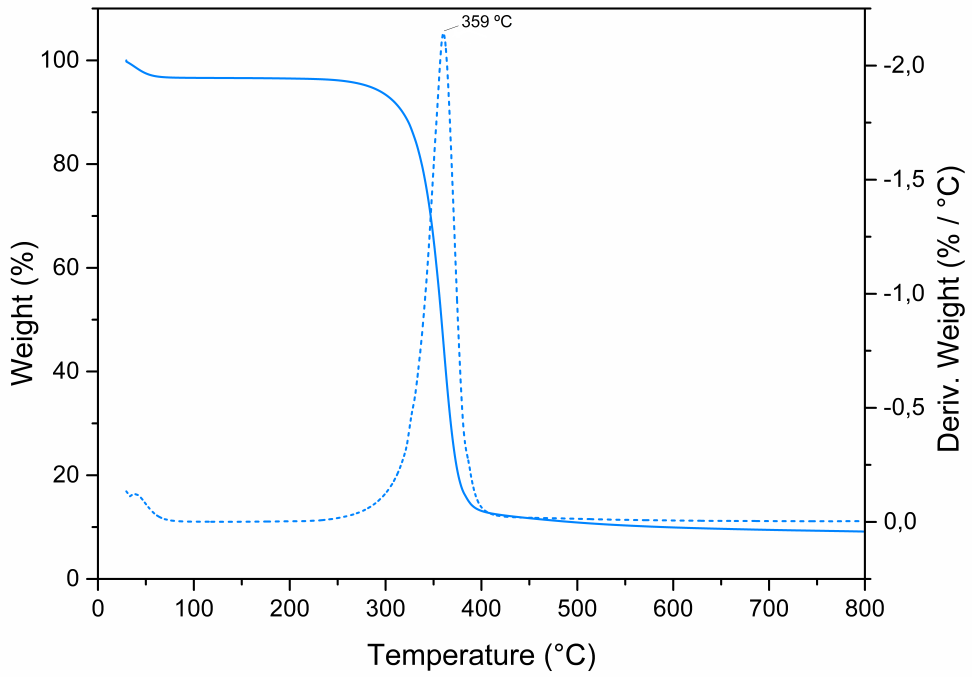

Figure S3: TGA curve (solid line) and DTG curve (dotted line) obtained for methylcellulose used as a binder in the composite formulations.

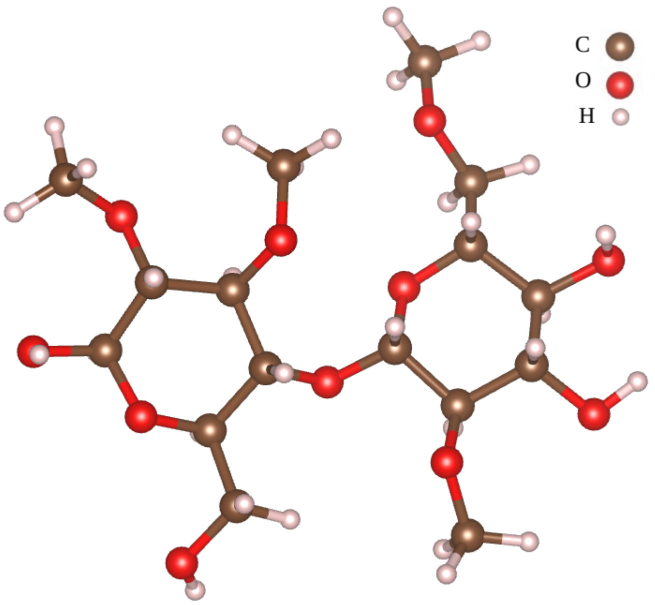

Figure S4: The optimized structure of the methylcellulose fragment utilized in this study

Table S1: MOF composite preparation conditions.

**Composite**	**MOF (g)**	**MC (g)**	**EtOH/H_2O (mL)***
MC/UiO-66	2.0245	0.1033	2.0
MC/MIL-53(Al)-TDC	2.0141	0.1006	2.0
MC/ZIF-8	2.0030	0.1030	0.8
MC/HKUST	2.0730	0.1029	1.0

* Concentration: 50/50 % (v/v).

Table S2: Crush strength of commercial materials, as specified by the suppliers

**Material**	**Supplier**	**Type**	**Main Use**	**Form**	**Size (mm)**	**Crush Strength (N)**
BJLL3-5	Ningxia Yongruida Carbon Co., Ltd.	Activated Carbon	Desulfurization and denitrification	Cylinder	3.0–5.0[a]	≥ 25
4A	Luoyang Jianlong Micro-Nano New Materials Co., Ltd	Zeolite	Dehydration	Cylinder	1.6–2.5[a]	≥ 40
JLOX-103	Luoyang Jalon Micro-Nano New Materials Co., Ltd.	Zeolite	Oxygen production	Sphere	–	≥ 15
5A-B1	Zhengzhou Gold Mountain Science and Technique Co. Ltd.	Zeolite	Petrochemical, hydrocarbon processing and air separation	Cylinder	2.0–2.8	≥ 30
13 X	Jiangxi OIM Chemical Co., Ltd ALL	Zeolite	Remove CO_2, moisture and desulfurization	Cylinder	1.6[a]	≥ 25/Pc[b]
JZ-ZHS	Shanghai Jiuzhou Chemicals Co. Ltd	Zeolite	Desulfurization and dehydration	Pellet	1.6	≥ 25/Pc[b]

[a] Cylinder diameter.
[b] Suppliers report the mechanical strength of each piece of molded material (Pc).

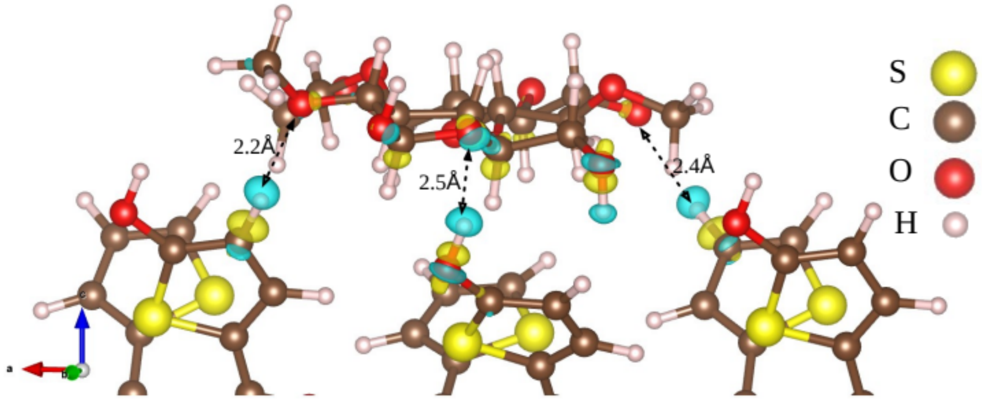

Figure S5: Charge density difference of (100) surface of MIL-53(Al)-TDC, with the closest distances between the surface and molecule. Yellow and cyan regions represent charge accumulation and depletion between the molecules, respectively. The isosurface contour is 0.003 e/Bohr^3.

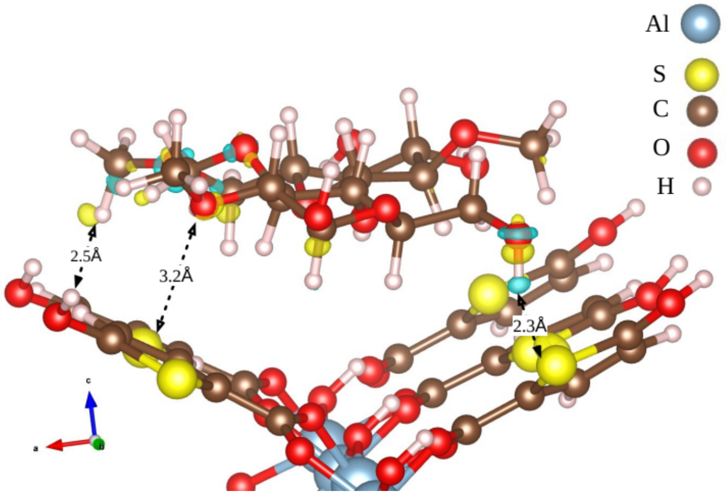

Figure S6: Charge density difference of (001) surface of MIL-53(Al)-TDC, with the closest distances between the surface and molecule. Yellow and cyan regions represent charge accumulation and depletion between the molecules, respectively. The isosurface contour is 0.003 e/Bohr^3.

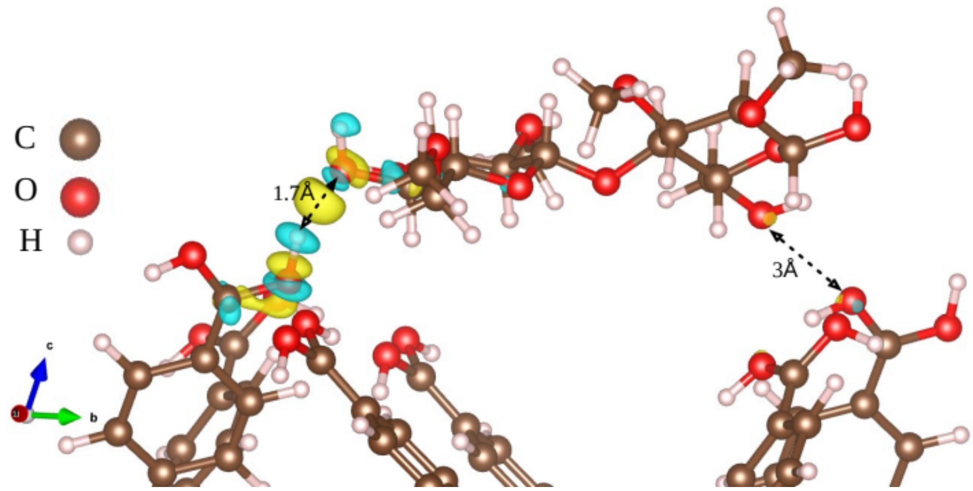

Figure S7: Charge density difference of (111) surface of UiO-66(Zr), with the closest distances between the surface and molecule. Yellow and cyan regions represent charge accumulation and depletion between the molecules, respectively. The isosurface contour is 0.003 e/Bohr^3.

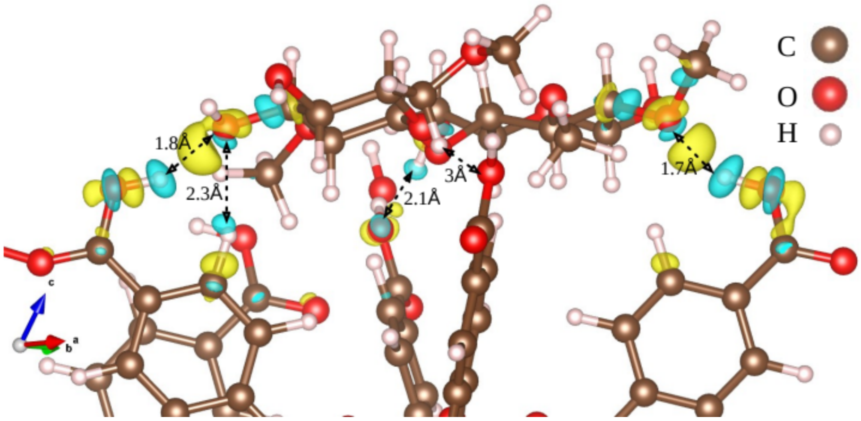

Figure S8: Charge density difference of (011) surface of UiO-66(Zr), with the closest distances between the surface and molecule. Yellow and cyan regions represent charge accumulation and depletion between the molecules, respectively. The isosurface contour is 0.003 e/Bohr^3.

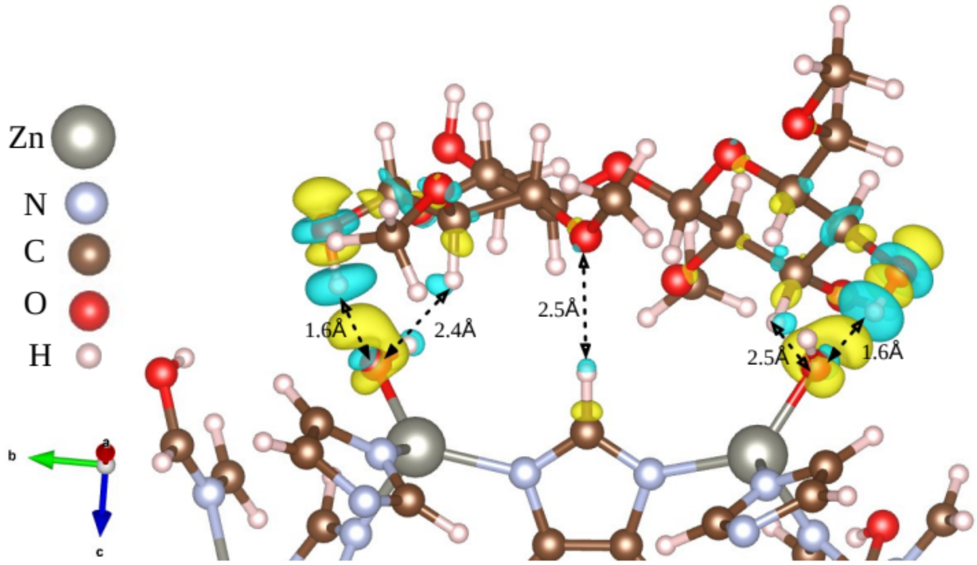

Figure S9: Charge density difference of (110) surface of ZIF-8, with the closest distances between the surface and molecule. Yellow and cyan regions represent charge accumulation and depletion between the molecules, respectively. The isosurface contour is 0.003 e/Bohr^3.

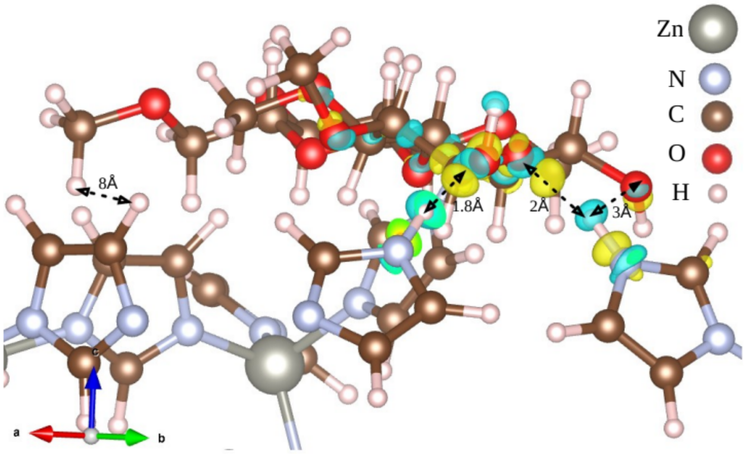

Figure S10: Charge density difference of (111) surface of ZIF-8, with the closest distances between the surface and molecule. Yellow and cyan regions represent charge accumulation and depletion between the molecules, respectively. The isosurface contour is 0.003 e/Bohr^3.

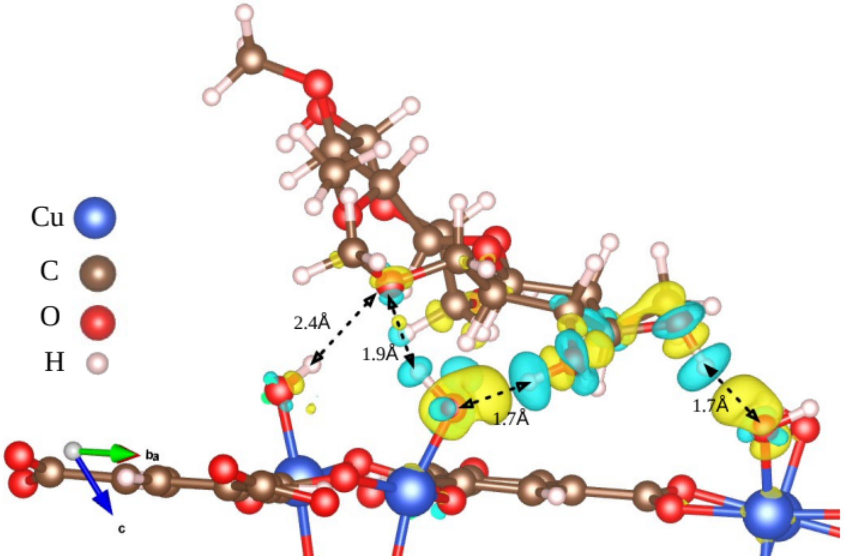

Figure S11: Charge density difference of (001) surface of HKUST, with the closest distances between the surface and molecule. Yellow and cyan regions represent charge accumulation and depletion between the molecules, respectively. The isosurface contour is 0.003 e/Bohr^3.

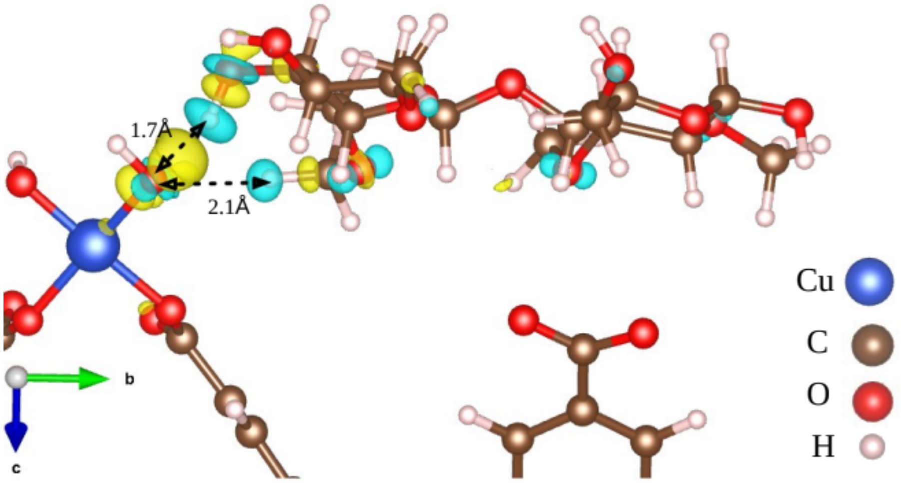

Figure S12: Charge density difference of (110) surface of HKUST, with the closest distances between the surface and molecule. Yellow and cyan regions represent charge accumulation and depletion between the molecules, respectively. The isosurface contour is 0.003 e/Bohr^3.

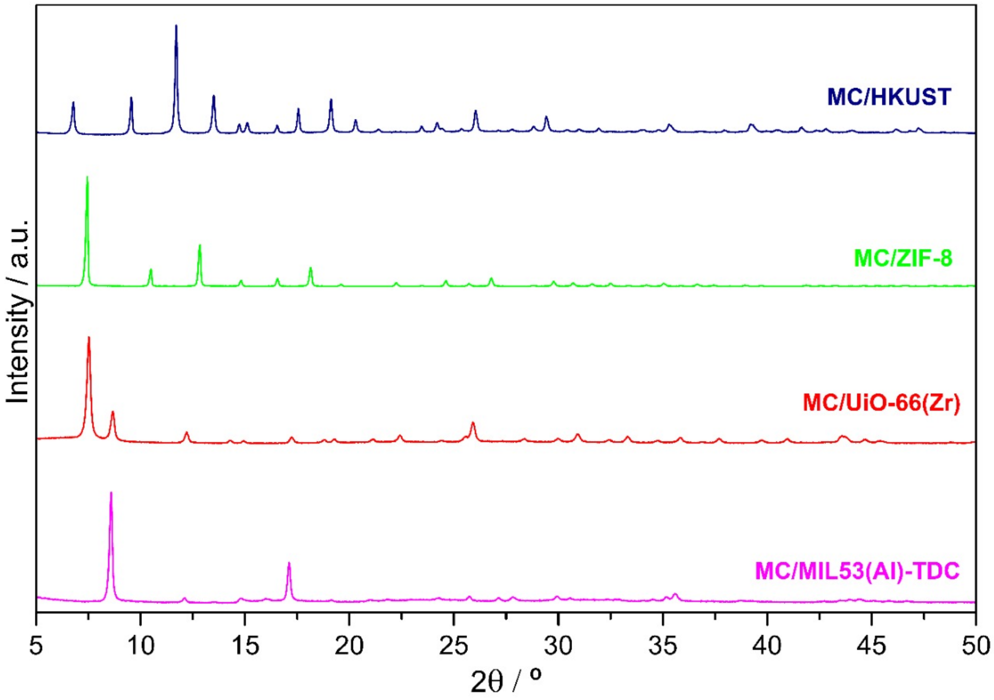

Figure S13: Experimental PXRD patterns of extruded composites activated in air at 100 ∘C and stored for more than six months: MC/HKUST (blue), MC/ZIF-8 (green), MC/UiO-66(Zr) (red), and MC/MIL-53(Al)-TDC (magenta).
